# Chloroplasts in C3 grasses move in response to blue-light

**DOI:** 10.1007/s00299-020-02567-3

**Published:** 2020-07-13

**Authors:** Weronika Krzeszowiec, Maria Novokreshchenova, Halina Gabryś

**Affiliations:** Department of Plant Biotechnology, Faculty of Biochemistry, Biophysics and Biotechnology, Jagiellonian University, Gronostajowa 7, 30-387 Kraków, Poland

**Keywords:** Blue light, Cereals, Brachypodium distachyon, Phototropins expression

## Abstract

**Key message:**

***Brachypodium distachyon*** **is a good model for studying chloropla st movements in the crop plants, wheat, rye and barley. The movements are activated only by blue light, similar to Arabidopsis.**

**Abstract:**

Chloroplast translocations are ubiquitous in photosynthetic organisms. On the one hand, they serve to optimize energy capture under limiting light, on the other hand, they minimize potential photodamage to the photosynthetic apparatus in excess light. In higher plants chloroplast movements are mediated by phototropins (phots), blue light receptors that also control other light acclimation responses. So far, *Arabidopsis thaliana* has been the main model for studying the mechanism of blue light signaling to chloroplast translocations in terrestrial plants. Here, we propose *Brachypodium distachyon* as a model in research into chloroplast movements in C3 cereals. Brachypodium chloroplasts respond to light in a similar way to those in Arabidopsis. The amino acid sequence of Brachypodium *PHOT1* is 79.3% identical, and that of *PHOT2* is 73.6% identical to the sequence of the corresponding phototropin in Arabidopsis. Both phototropin1 and 2 are expressed in Brachypodium, as shown using quantitative real-time PCR. Intriguingly, the light-expression pattern of *BradiPHOT1* and *BradiPHOT2* is the opposite of that for Arabidopsis phototropins, suggesting potential unique light signaling in C3 grasses. To investigate if Brachypodium is a good model for studying grass chloroplast movements we analyzed these movements in the leaves of three C3 crop grasses, namely wheat, rye and barley. Similarly to Brachypodium, chloroplasts only respond to blue light in all these species.

**Electronic supplementary material:**

The online version of this article (10.1007/s00299-020-02567-3) contains supplementary material, which is available to authorized users.

## Introduction

It is a truism to say that the increasing demand for global food production is one of the challenges for plant molecular biology these days. Various models that span from cellular metabolism to redesigning a whole canopy have been discussed to improve crop productivity (Ort et al. 2015). One interesting aspect that has been ignored in this discussion is light-directed chloroplast positioning, ubiquitous in mesophyll cells. Chloroplast movements protect photosynthetic apparatus in high light (Kasahara et al. 2002; Sztatelman et al. 2010) and enhance photosynthesis in low light (Zurzycki 1955). Moreover, rearrangements of chloroplasts in high light result in a reduction in the leaf internal CO_2_ conductance (Tholen et al. 2008). As a consequence, changes in the position of chloroplasts can limit the rate of photosynthesis. Recently, it has been suggested that the process of chloroplast movements is sensitive to the relative water status of the cell (Nauš et al. 2016). Although a crosstalk between chloroplast movements and drought stress is an interesting possibility, this idea needs further investigation. All the above information leads to the conclusion that further understanding of the chloroplast relocation mechanism might contribute to the improvement of photosynthetic productivity of crop plants.

Chloroplast movements depend on the direction, wavelength and intensity of light. Notably, they are restricted to illuminated cells. In most species analyzed so far, chloroplasts relocate in blue light according to two distinct mechanisms depending on the fluence rate. Weak blue light causes relocation of chloroplasts toward the most illuminated cell walls (accumulation response), while strong blue light causes chloroplasts to gather at the cell walls parallel to the light direction (avoidance response). In *Arabidopsis thaliana* the photoreceptors involved in the movements are phototropin1 (phot1) and phototropin2 (phot2). Both phot1 and phot2 control the accumulation response (Sakai et al. 2001), but only phot2 controls the avoidance response (Jarillo et al. 2001). Phototropins are blue/UV-A photoreceptors that contain a C-terminal serine-threonine kinase domain and two LOV (light, oxygen, voltage -regulated) domains. FMNs bound to LOV domains function as chromophores. FMN/LOV domains activated by blue-light bring about autophosphorylation of kinase domains (for more details see review: Banaś et al. 2012). Although phototropins are hydrophilic and contain no obvious membrane-spanning domains they are associated with the cell membrane. They have been observed to cycle in the cell and this trafficking is believed to participate in their function (Sakamoto and Briggs 2002; Aggarwal et al. 2014). However, the role of phototropin cycling as a part of their functioning has recently been questioned (Liscum 2016). In addition to chloroplast redistribution, phototropins control other acclimation movements including phototropism (Sakai et al. 2001), stomatal opening (Kinoshita et al. 2001), nuclear avoidance movement (Iwabuchi et al. 2007) and leaf flattening (Inoue et al. 2008).

The exact signaling pathway from phototropins to chloroplast positioning is yet to be determined. Phospholipase C has been demonstrated to play a role in phot2 signaling in avoidance movements of Arabidopsis chloroplasts, while PI3K and PI4K are required for the accumulation response of chloroplasts mediated by both phototropins (Aggarwal et al. 2013). Thus, the phosphoinositide-calcium pathway is involved in the chloroplast movement mechanism (Łabuz et al. 2016). However, calcium channels contributing to this signaling have not been identified.

The ability of plant cells to relocate chloroplasts upon blue-light irradiation is widespread amongst different taxa (Gabryś and Krzeszowiec 2012). The species studied so far belong to *Chlorophyta*, *Charophyta*, *Bryophyta*, *Lycopodiopsida*, *Pteridophyta*, *Angiosperms* and *Gymnosperms*, although only a few representatives of these systematic groups have been studied. In Angiosperms, most available data concern two dicotyledonous model plants, *Arabidopsis* and *Nicotiana*. Among monocot species *Vallisneria* sp., *Tradescantia albiflora* and *Lemna trisuca* have been investigated (review Gabryś and Krzeszowiec 2012). Surprisingly, for many decades chloroplast movements have not been investigated in grasses, and in particular in cereals, in spite of their crucial agricultural importance. The first reports showing rearrangement of chloroplasts in cereals analyzed finger millet and sorghum (Maai et al. 2011, 2019). Recently, avoidance movement in barley has also been reported (Nauš et al. 2016).

Here, we show blue light-directed chloroplast redistribution in the leaves of three agriculturally important crop species, namely wheat, rye and barley and of a C3 grass *Brachypodium distachyon*. To facilitate the research on the mechanism of chloroplast movements in temperate zone cereals, we propose to use Brachypodium as a model plant. An advantage of Brachypodium is that, due to its small genome, it is amenable to genetic transformation.

## Materials and methods

### Plant materials and growth conditions

The *B. distachyon* seeds were a kind gift of prof. R. Hasterok (Silesia University, Poland). The wheat (*Triticum aestivum*), rye (*Secale cereale*) and barley (*Hordeum vulgare*) seeds were obtained from a seed commercial store (Kraków, Poland). The seeds were soaked for 24 h in 3 mM solution of KMnO_4_, which provided efficient disinfection. After washing in tap water, they were transferred to a wet tissue and grown in darkness for 5–7 days. The etiolated seedlings of about 2–4 cm in height were transferred to commercial soil (Compo Sana, Compo Expert) mixed with vermiculite, 3:1 (Vermiculite Poland Ltd.).

For the chloroplast movement investigations all four species were grown in a glasshouse, at 23 ± 3 °C, with additional light provided by a 400 W HMI light bulb (HQI-BT 400 W/D Pro Daylight E40), at the photoperiod of 14L/10D. On a sunny day PPFD was 100–200 µmol m^−2^ s^−1^ at the level of the leaves. The experiments were performed on 4–6 week old plants.

For expression studies Brachypodium plants were soil-grown in a growth chamber (Sanyo MLR-350H) at the photoperiod of 23 ± 2 °C, 14L/10D photoperiod, and illuminated with fluorescent lamps (Philips Master TL-D 36 W/840, Osram L36 W/77 Fluora, Activa 172-36W, Sylvania Gro-Lux F36W/GRO-T8) with an average PPFD of 110 μmol m^−2^ s^−1^.

### Photometric measurements of chloroplast movements

Quantitative measurements of chloroplast movements were performed on ca. 0.8 cm long leaf segments using a double-beam photometer (Gabryś et al. 2017). A red light of 660 nm, 0.1 μmol m^−2^ s^−1^, modulated with a frequency of 800 Hz was used to monitor changes in the transmittance through the leaf. A blue light of 460 nm (Luxeon Royal Blue LXHL-FR5C diode, Philips Lumiled Lighting Comp., San Jose, CA, USA) caused chloroplast redistributions. Plants were dark-adapted overnight for at least 12 h prior to the experiments. After recording the initial transmittance level, leaf segments were illuminated with weak blue light (1.6 μmol m^−2^ s^−1^) for 45 min followed by strong blue light (108 μmol m^−2^ s^−1^) for the same time. The following parameters were measured/calculated for both responses: (1) amplitude—the transmittance change after 45 min, (2) velocity—the first derivative of the initial linear fragment of the transmittance curve (for details see Gabryś et al. 2017). To measure the fluence rate response curves chloroplast relocations were induced by a continuous blue light of fluence rates increasing stepwise, each step lasting 45 min. The chloroplast movements of four species grown in the same growing conditions were compared. The movements were measured in the mature leaves of fully grown plants in the vegetative phase (before any sign of flowering). Whenever it was possible, leaves with similar initial transmittance levels were chosen for the experiment.

To image chloroplast movements in grasses we cut leaf blades into pieces which were several mm in length. The lower epidermis was partially removed and water was introduced into intracellular spaces under weak negative pressure. These infiltrated leaf fragments were placed on a slide in a drop of water and irradiated in the photometer with the simultaneous measurement of the transmittance level. Photographs were taken using MW 50 Series Microscope (Opta-Tech Ltd.) microscope equipped with HDMI camera (Opta-Tech Ltd.) after transmittance reached a stationary minimum or maximum levels.

### Light treatments in experiments to investigate phototropin expression

Leaves from 4-week-old plants were collected to determine whether phototropin expression is blue or red light controlled. Plants were dark-adapted for 16 h prior to irradiation. Because *PHOT1* is a clock regulated gene in Arabidopsis (see *NPH1* in Harmer et al. 2000), irradiations were always started at 10 am and finished at 1 pm ± 15 min. 3 leaves were detached from 3 different plants and placed on wet tissue paper to prevent drying. Dark-control leaves were cut off at the same time as the irradiated ones, and kept in darkness on wet tissue paper till 1 pm. Leaves were irradiated with blue or red light of 36 μmol m^−2^ s^−1^ (strong irradiation) or 2 μmol m^−2^ s^−1^ (weak irradiation) for 3 h. Blue light was obtained from LXHL-PR09 LEDs (Ledium Ltd., Hungary) with a maximum emission at 455 nm and a half-band width of 20 nm. Red light was obtained from Luxeon Rebel ES LEDs (Philips Lumileds Lighting Comp.) with a maximum emission at 655 nm and a half-band width of 14 nm. Following 3 h long light treatment, the exposed and dark-adapted leaves were immediately frozen in liquid nitrogen.

### Expression of phototropins

For real time PCR *B. distachyon* leaves were harvested from 4-week-old plants. The total RNA was extracted using RNeasy plant mini kits (Qiagen) with modifications as described in Kurbidaeva et al. (2014). For real time PCR, first-strand cDNA synthesis was performed using the Fermentas RevertAid™ First Strand cDNA Synthesis Kit with Oligo_dT primers according to the manufacturer’s instructions, using a 20 µL reaction volume and an incubation time of 1 h at 42 °C with total RNA of either 0.1 or 0.01 mg. The cDNA reaction mixture was diluted tenfold with water, and 2 µL were used as a template in a 20 µL PCR reaction using the Applied Biosystems FAST 7500 real-time PCR system in standard mode with SYBR Green PCR CoreReagents Mix (Applied Biosystems) according to the manufacturer’s instructions. For gene transcript analysis the annealing/extension temperature was 58/62 °C. The reactions were carried out in triplicate and products checked using melting curve analysis. The abundance of transcripts was analyzed with the relative standard curve method (Delta-delta Ct method), normalizing to the reference gene Ubi4. The reference gene was chosen according to the technical approach used in previous Brachypodium studies (Hong et al. 2008; Chambers et al. 2012). The primers used for amplification are shown in Table 1. The experiment was performed in three biological replicates.

**Table 1:** List of primers used in this study

Name	Sequence	Tm	Origin
MGN_RT_Phot1_L	CTCTTTCCAGACGACATGAGG	59	Own design
MGN_RT_Phot1_R	GGTTATCATCACCACCACCAG	60	Own design
MGN_RT_Phot2_L	TAATTGGCAAGGAATCTCAACC	60	Own design
MGN_RT_Phot2_R	AAGCACACCTTCCTCAGGATT	60	Own design
MGN_RT_UBI4_L	TGACACCATCGACAACGTGA	60	Hong et al. (2008)
MGN_RT_UBI4_R	GAGGGTGGACTCCTTCTGGA	60	Hong et al. (2008)
MGN_RT_UBQ10_L	TGGACCCTACAATCTGTTTGC	60	Own design
MGN_RT_UBQ10_R	CAGTTAAGTGGGCTGTCTGCT	59	Own design

An attempt was made to compare the *BrachPHOT1* and *BrachPHOT2* mRNA levels with the protein levels evaluated by western blot. We used antibodies for Arabidopsis Anti-PHOT1 (AS10 720) and anti-PHOT2 (AS10 721) purchased from (Agrisera), detailed information in Łabuz et al. (2015) and Sztatelman et al. (2016). The experiment was repeated several times. Unfortunately the antibodies did not recognize Brachypodium phototropins. Antibodies had been raised against short synthetic peptides from N-terminal phototropin fragments. The PHOT1 peptide (NH2)CKPQKSAVAAEQRAA(CONH2) covered amino acids 105–118 of the protein, the PHOT2 peptide(NH2)CSSKWMEFQDSAKIT(CONH2) covered amino acids 54–67. These antibodies are apparently not suitable for Brachypodium.

### *Cis*-element identification and microarray analysis

Brachypodium TF binding sites and signal sequences were obtained using a web-based tool from A Database of Plant Cis-acting Regulatory DNA Elements (PLACE) (Higo et al. 1999) (http://www.dna.affrc.go.jp/PLACE/). In this study, we only used those predicted cis-elements located within 1 kb region upstream of the *PHOT1* and *PHOT2* start codon.

### Sequence retrieval and phylogenetic analysis

For the reconstruction of the phylogenetic tree, potential orthologs of the *PHOT1* and *PHOT2* genes of Arabidopsis were identified using multiple database searches. Protein sequences of the Phot1(At) and Phot2(At) were used as a query in BLASTP (Altschul et al. 1990), searches against the public databases Phytozome v12.1 (http://www.phytozome.net/) and GenBank (http://www.ncbi.nlm.nih.gov/BLAST/) using the default settings. Protein sequences with an expect value ≤ 1e−05 were retrieved and redundancies removed. Sequence alignment was generated with MUSCLE (Thompson et al. 1994). A conserved protein sequence was identified within the PAS domain and sequences downstream of it was selected for analysis. The phylogenetic relationships of the trimmed *PHOT* genes were reconstructed using the maximum likelihood method with MEGA X (Kumar et al. 2018). Maximum likelihood analyses were performed using a Poisson substitution model, gamma distribution of mutation rates among sites, and no gap deletion. Support for each node was tested with 100 bootstrap replicates for both procedures. As outgroups, we used PHOT1 or PHOT2 protein sequences form *Sellaginella moelendorfii*. The accession numbers of all the sequences used are presented in Supplementary Tab. S1.

## Results

### Chloroplast movements in grasses

Two main types of photoreceptors, phototropins and phytochromes participate in the control of chloroplast movements among plant taxa. Red and blue irradiations were used to find out which of them (or perhaps both?) is involved. First, the light transmittance of leaves irradiated with continuous red light (80 μmol m^−2^ s^−1^) was measured for 90 min. No transmittance changes were detected in these conditions (Fig. S1). The results show that phytochromes alone do not activate directional chloroplast movements in the tested species.

**Fig. 1:**
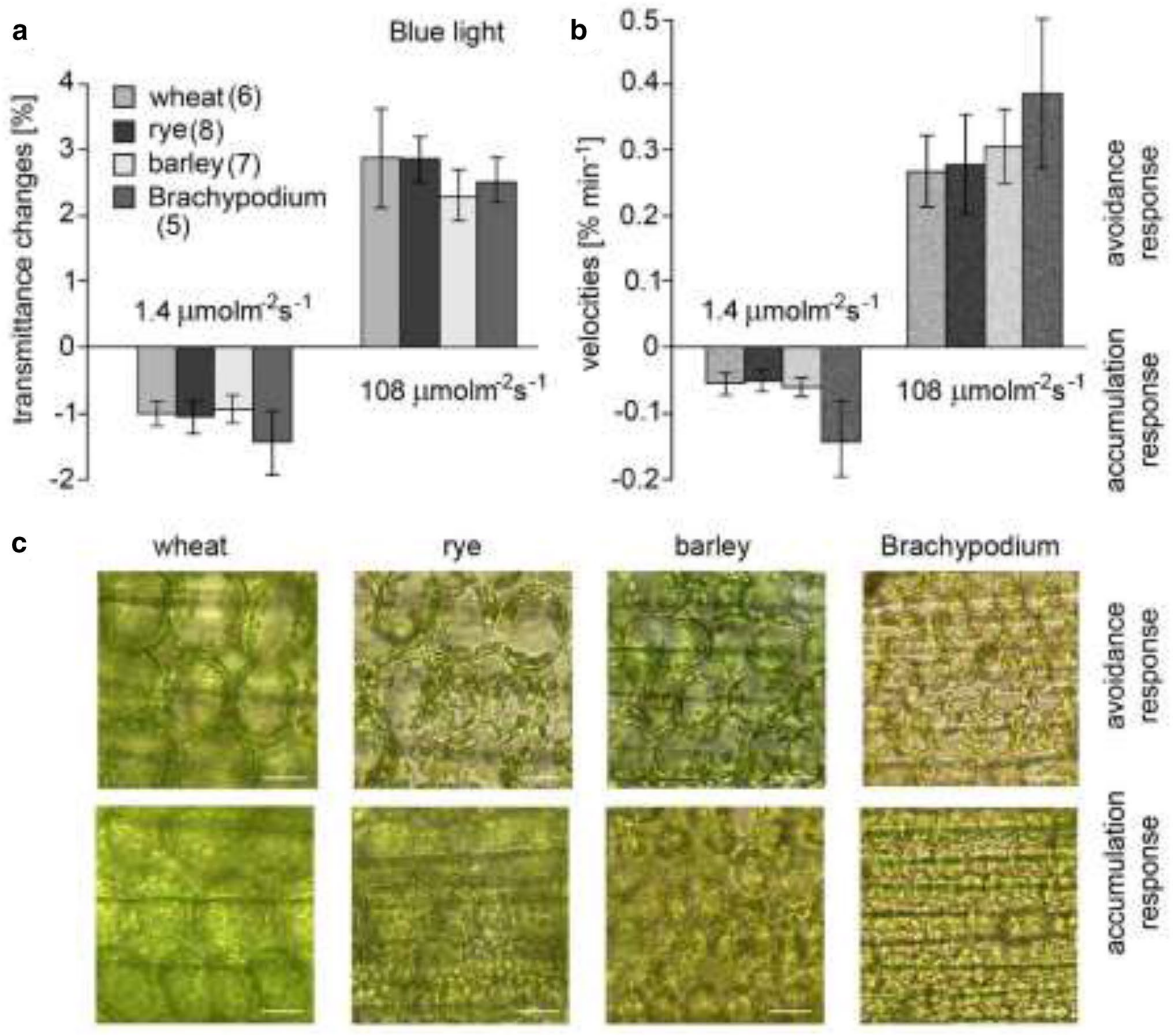
Parameters of transmittance changes reflecting chloroplast relocations induced by continuous blue light in four species, wheat (*Triticum aestivum*), rye (*Secale cereal*), barley (*Hordeum vulgare*) and *Brachypodium distachyon*a). **a** Amplitudes of transmittance changes reflecting chloroplast positions after 45 min of continuous blue light; **b** velocities of transmittance changes reflecting chloroplast relocations in continuous blue light. The results are the means of 5–8 experiments; number of replicates is given in brackets. Error bars represent standard deviations. **c** Light microscopy images of tissues after 1 h of blue light irradiation (108 μmol m^−2^ s^−1^—upper row, or 1.4 μmol m^−2^ s^−1^—lower row). Scale bars 20 μm

In contrast to red light, blue light-activated directional chloroplast relocations in all the species studied. Weak blue-irradiation (1.4 μmol m^−2^ s^−1^) caused the accumulation response of chloroplasts while irradiation with strong blue light (108 μmol m^−2^ s^−1^) caused the avoidance response. Figure 1c demonstrates the stationary positions of chloroplasts attained in the accumulation and avoidance responses for the tested species. The respective mean transmittance changes are shown in Fig. 1a. In weak light, the biggest changes in amplitudes occurred in Brachypodium followed by rye, wheat and barley. The amplitudes of avoidance responses activated by strong light were more uniform. The respective velocities of transmittance changes are shown in Fig. 1b.

**Fig. 2:**
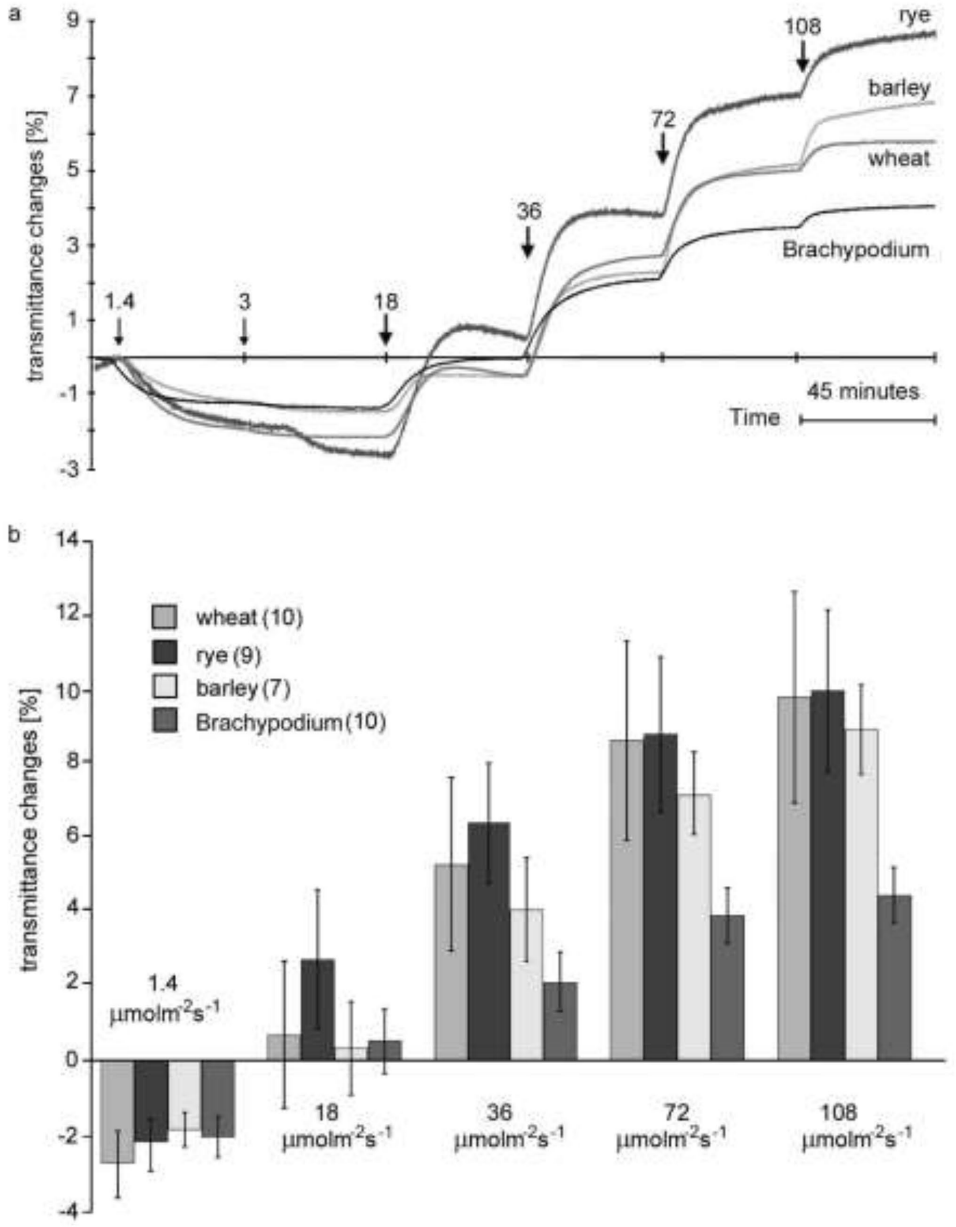
Fluence rate response curves and mean transmittance changes. Responses of chloroplasts in leaves of wheat, rye, barley and *Brachypodium distachyon* to low and high intensities of continuous blue light measured as a percentage transmission at 660 nm as a function of time. **a** Representative fluence rate response curves; **b** mean leaf transmittance changes measured after consecutive steps in fluence rates. Number of replicates is given in brackets

To obtain further insight into chloroplast responses fluence rate response curves were measured. Leaves were irradiated with blue light increasing stepwise from 1.4 to 108 μmol m^−2^ s^−1^. Successive increases in fluence rates led to changes in chloroplast positioning, and steady-state transmittance levels were attained for each step. Fluence rate response curves, representative of the biological replicates analyzed are shown in Fig. 2a. The means of transmittance changes following 45 min exposures to light are presented in Fig. 2b. In that experimental series the smallest changes were recorded for Brachypodium, consistent with the lowest initial transmittances measured after dark adaptation. The recorded average initial transmittance was equal to 14.1% for Brachypodium, and was higher for the cereals: 16.8, 20.7 and 24.4% for wheat, rye and barley, respectively. The lowest dark transmittance measured for Brachypodium reflected the highest optical density of the leaves. Consequently, the transmittance changes measured were also the lowest for this grass, although their ratios were similar to other species. It should be stressed that the values of light transmittance through leaves vary. The optical properties of leaves depend on light conditions during growth, among other things. In the second experimental series, Brachypodium leaves were optically denser than those of cereals measured and, as a consequence, the transmittance changes were lesser than these measured for other species. Importantly, the ratios of accumulation to avoidance amplitudes were comparable among the species tested.

The statistical analysis of the obtained parameters is given in Fig.S2. To enable the statistical comparison between species, transmittance amplitudes at consecutive fluence rate steps were normalized to the maximum transmittance changes corresponding to full accumulation and avoidance responses for individual leaves.

### Comparison of phototropins in Brachypodium and Arabidopsis

In order to further investigate light signaling in Brachypodium we performed a bioinformatics analysis of phototropin sequences and their promoter regions in this species. Homologues of Arabidopsis phototropins in the Brachypodium genome were selected on the basis of maximum identity in the Phytozome12 database. *AtPhot1* (AT3G45780) corresponds to the orthologue Bradi4G45310 (*BradiPhot1*) with 79.3% identity (*E* value = 1.7E−27), and the Bradi5G07360 (*BradiPhot2*) gene is orthologous to *AtPhot2* (AT5G58140) with 73.6% identity (*E* value = 7E−26).

To characterize the phototropin orthologues more accurately, a comparative analysis of the promoters for all four genes was carried out (Supplementary Tab 2). The assessment revealed promoter similarities in *AtPhot1*, *AtPhot2*, *BradiPhot1* and *BradiPhot2*. Fifteen motifs potentially relevant to light regulation have been found to occur at least once in these promoter regions. Seven motifs have been found in all analyzed promoters. Eight motifs are different in Arabidopsis and Brachypodium promoters: (1) ASF1MOTIFCAMV is present in *AtPhot2, BradiPhot1* and *BradiPhot2*, (2) REALPHALGLHCB21 is present in *AtPhot1, BradiPhot1 and BradiPhot2*, (3) TBOXATGAPB is specific for *BradiPhot2* and absent elsewhere, (4) SORLIP1AT is present in *AtPhot2* and *BradiPhot2*, (5) SORLIP2AT is present in *AtPhot1* and *BradiPhot2*, (6) SORLIP5AT is present only in *AtPhot1*, (7) SORLREP2AT is present only in *BradiPhot2*, (8) PRECONSCRHSP70A is present only in both Brachypodium promoters.

**Fig. 3:**
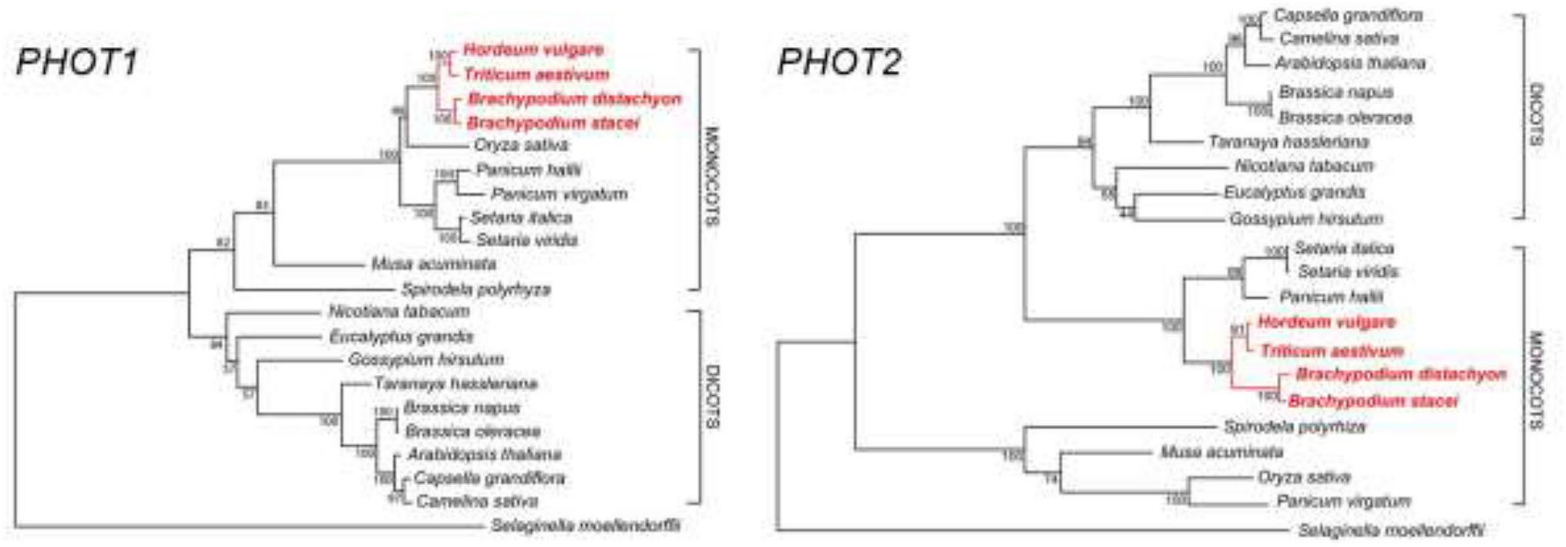
Phylogenetic analysis of *PHOT1* and *PHOT2* sequences in a selection of monocot and dicot species. Red color denotes species for which phototropin has the highest degree of homology to that of the model plant Brachypodium and crop species, barley and wheat. Bootstrap values (1–100) are given at each branch (colour figure online)

In order to investigate if Brachypodium is a suitable model for studying light signaling in grasses we wanted to compare monocot and dicot phototropin sequences. To this end, we constructed phylogenetic trees of PHOT1 and PHOT2 orthologues using the maximum likelihood method (Fig. 3). The cladograms are drawn to scale, with branch lengths corresponding to the number of substitutions per site. Our phylogenetic analysis indicates that Brachypodium PHOT1 and PHOT2 sequences are most similar to these of *H. vulgare* and *T. aestivum*. The high degree of homology of cereal and Brachypodium phototropin sequences suggests that the mechanism of blue light signaling may be similar in these species. We were unable to identify phototropin sequences for rye using Phytozome or Genebank databases.

### Expression of phototropins in Brachypodium

**Fig. 4:**
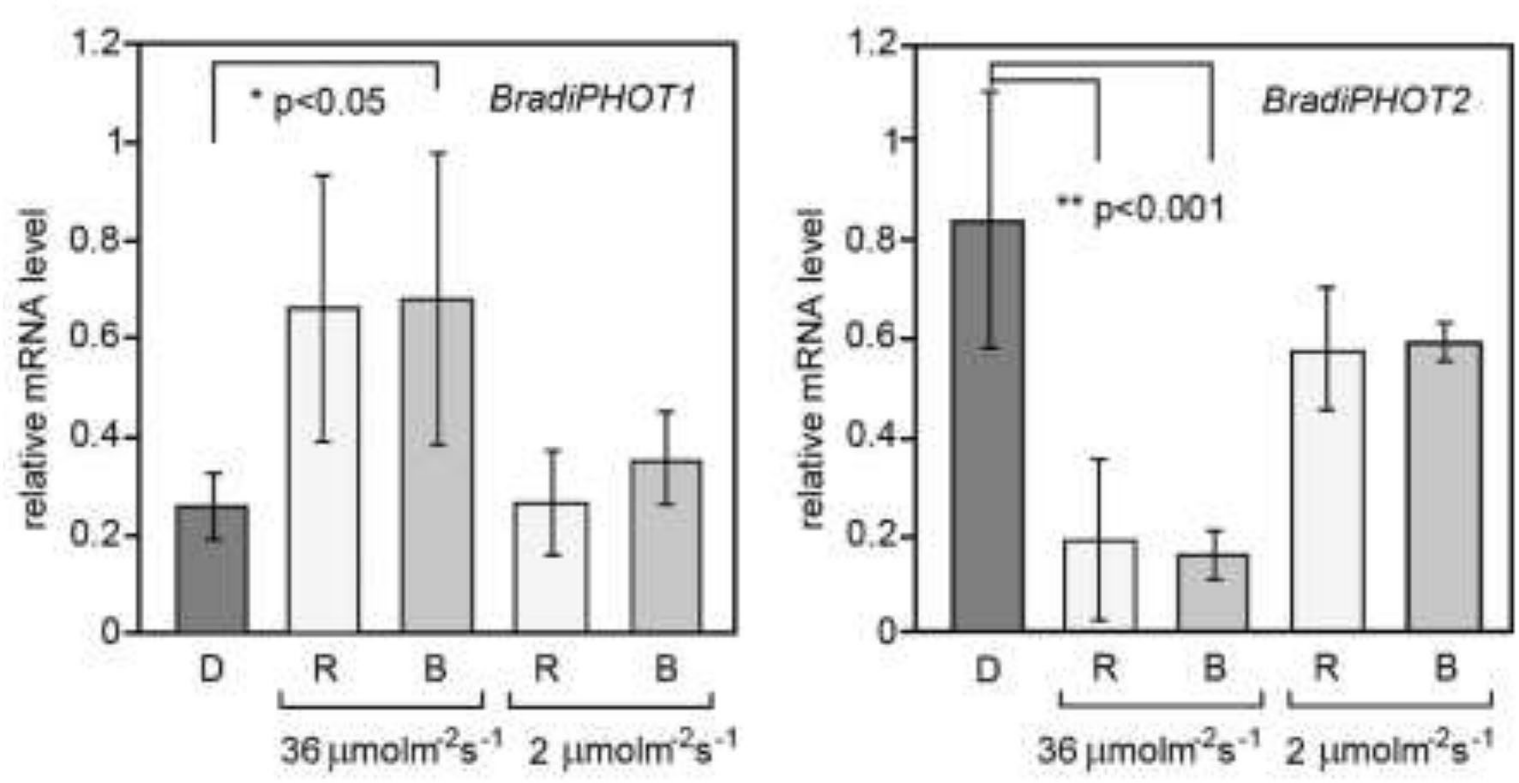
The expression of *BradiPHOT1* and *BradiPHOT2* in Brachypodium leaves irradiated with blue or red light of different fluence rates. The relative mRNA levels found in leaves of dark-adapted plants and plants irradiated for 3 h. Strong red (SR), strong blue (SB), weak red (wR) or weak blue (wB) light treatments were used. Strong light was equal to 36 µmol m^−2^ s^−1^ and weak light had a fluence rate of 2 μmol m^−2^ s^−1^. Each column represents the mean of three biological replicates for mRNA isolated from a pool of three leaves detached from three plants. Error bars indicate the standard error. Asterisks indicate the statistical significance of the difference between dark- and light-treated samples

Given that red light does not induce chloroplast movements in grasses (Fig S1), phototropins are most probably the only photoreceptors involved in blue-light driven chloroplast reorganization in *B. distachyon*. In general, the level of *BradiPHOT1* was lower than that of *BradiPHOT2* in dark-adapted plants. Strong blue or red light upregulated the *BradiPHOT1* gene, while weak light did not change its transcription level independently of the wavelength. In the case of *BradiPHOT2* the expression was downregulated by strong light (Fig. 4).

## Discussion

The first extensive monograph about chloroplast movements was published at the beginning of the twentieth century (Senn 1908). Twenty-four years ago, the movements were characterized in *A. thaliana* (Trojan and Gabrys 1996). Since then most of the work on chloroplast movements—e.g., the discovery of the main photoreceptors (Christie et al. 1998; Jarillo et al. 2001)—has been carried out using this model plant. For unknown reasons, chloroplast movements in economically important crop species attracted much research effort. In particular, the concept that chloroplast movements serve to improve photosynthetic efficiency has never been supported by data on crop species. Thus, this paper serves two purposes. Firstly, we demonstrate chloroplast movement responses to weak and strong blue light in C3 crop grasses. Secondly, we introduce a working hypothesis that *B. distachyon* is a good model plant for further studies on these movements in crop grasses.

Four cereal crops have been shown so far to exhibit chloroplast movements. Previously published data concerned three C4 grasses, namely finger millet, maize and sorgo, and a single C3 species—barley. The chloroplasts have been shown to redistribute in response to white light in finger millet (*Eleusine coracana*) and maize (*Zea mays*) (Yamada et al. 2009). Notably, the movements were found only in mesophyll cells, never in the bundle sheath. In another study abscissic acid was shown to shift blue light-induced avoidance movements to aggregative movements toward the bundle sheath side, in both finger millet and maize (Maai et al. 2011). In Arabidopsis, no impact of exogenous ABA on the amplitudes and kinetics of chloroplast movements was detected (Eckstein et al. 2016). ABA is not directly involved in phototropin-controlled chloroplast responses in mature Arabidopsis leaves. However, the disturbance of ABA biosynthesis and signaling in *aba1* and *abi2* mutants affects some elements of the chloroplast movement mechanism. In line with its role as a stress hormone, ABA appears to enhance plant sensitivity to light and promote the chloroplast avoidance response (Eckstein et al. 2016). While Maai et al. (2011) were unable to show chloroplast redistribution in barley (*H. vulgare*), avoidance movement in this species was reported 5 years later (Nauš et al. 2016). According to our results both accumulation and avoidance responses are conspicuous in barley and in other C3 crop species that we have analyzed. To explain these contradictory results we hypothesize that fluence rate during growth may affect the chloroplast relocation system in barley. The plants that did not show chloroplast movements (Maai et al. 2011) were grown at PAR 4–5 times stronger than those in which the movements took place (Nauš et al. 2016 and the present work). This explanation needs to be carefully validated as no correlation was found between excess light tolerance and the extent of chloroplast moments in several monocot and dicot species (Königer and Bollinger 2012).

Recently, chloroplast movements have also been shown in Sorghum (*Sorghum bicolor*) (Maai et al. 2019). The experiments concerned midday depression of photosynthesis and chloroplast positioning in excess light. The avoidance response of chloroplasts was shown to appear concomitantly with depression in the net photosynthesis rate. Although high fluence rate of 1200 μmol m^−2^ s^−1^ was used the experiments provide a new and interesting link between chloroplast positioning and photosynthesis.

So far, photoreceptors that activate chloroplast relocation in grasses have not been identified. In the four species under investigation chloroplast movements are blue-light directed (Figs. 1, 2) and no responses of chloroplasts to red light were found (Fig.S1). Thus we assume that the movements are controlled by phototropins in these grasses.

Red light absorbing photoreceptors control chloroplast relocation in several species. In the monocot water angiosperm *Vallisneria gigantea* the relocation is mediated by phytochrome/photosynthesis in red light (Izutani et al. 1990), and, most probably, by phototropins in strong blue light (Sakai et al. 2015, Sakurai et al. 2005). The fern *Adiatum capillus veneris* contains a chimeric photoreceptor, neochrome1 (former name phytochrome3) made up of the chromophore-binding domain of phytochrome and nearly full-length phototropin1 (Kawai et al. 2003). Neochrome functions both as a red and a blue light receptor. However, independently of neochrome1 red light chloroplast relocation was also shown in the Adiantum mutant *neo1* (Sugiyama and Kadota 2011).

Chloroplast relocations in all dicot angiosperms studied so far are solely blue-light driven. Direct evidence that phototropins control chloroplast movements has only been obtained for the model plant *A. thaliana* due to the use of two null phototropin mutants *phot1* and *phot2* (Jarillo et al. 2001, Sakai et al. 2001). The responses in other higher land plants e.g., *Nicotiana tabacum*, *T. albiflora*, *Ajuga reptans*, and in the grasses investigated here are very similar both in terms of the extent and the kinetic of movement. The saturation of low-fluence-rate responses was about 1.4 μmol m^−2^ s^−1^. The high-fluence-rate response started near 18 μmol m^−2^ s^−1^ and complete high-fluence-rate rearrangement was around 108 μmol m^−2^ s^−1^. Irradiation with intermediate fluence rates (18, 36, 72 µmol m^−2^ s^−1^) produced incomplete avoidance responses. This supports our view that similar photoreceptors are active in all the cereals being investigated and most probably they are phototropins as in Arabidopsis.

The fluence rate response curves for all grasses under investigation (Fig. 2) were similar to those obtained for *A. thaliana, L. trisulca* and *T. albiflora* (Trojan and Gabrys 1996; Walczak and Gabrys 1980). It seems rather improbable that very similar curves obtained for the multilayer tissue of mature leaves reflect completely different molecular mechanisms.

We found a high level of similarity between the predicted amino acid sequence of *PHOT1* and *PHOT2* genes in Brachypodium and Arabidopsis. The sequence similarity between these photoreceptors in *B. distachyon*, *Brachypodium stacei*, wheat and barley is also high. Phylogenetic analyses indicate that the four species always form one clade. In our opinion this is another argument in support of Brachypodium as a model plant for studying chloroplast movements in C3 cereals.

Over a dozen proteins have been shown to participate in the signaling pathway downstream of photoreceptors. Table 2 shows a list of proteins found in several screens for chloroplast movement impaired mutants in Arabidopsis. Null mutants of all these genes show a reduction or inhibition in at least one chloroplast response. Most of them are believed to participate in the actin cytoskeleton regulation. A detailed analysis of their involvement in the movements is given in Banaś et al. (2012) and Kong and Wada (2016). To further validate Brachypodium as a model grass for chloroplast movement research, we investigated whether the protein toolbox engaged in chloroplast movements is similar in Arabidopsis and Brachypodium. The investigation was conducted using TAIR (The Arabidopsis Information Resource) and pBLAST (Protein Basic Local Alignment Search Tool). Null mutants of all genes shown in Table 2 have impaired chloroplast movements. The highest identity was observed for *PHOTs* and for *PP2A* (above 70% and 92%, respectively). The other genes show an identity ranging from 31% for *PMI2* to 64% for *KAC2*. An exception was found for *KAC* genes—only one sequence was found in Brachypodium. Our analysis indicates that the molecular machinery likely to be involved in the control of chloroplast movements is conserved in Brachypodium. The similarity to Arabidopsis may be useful for dissecting the signaling pathway in this species.

**Table 2:** Genes encoding proteins involved in the signaling pathway of chloroplast movements in *Arabidopsis thaliana* downstream of phototropins, and their homologs in *Brachypodium distachyon* found by NCBI/BlastP search

*Arabidopsis thaliana* gene	Role of a protein in chloroplast movements in *Arabidopsis*	Homolog gene in *Brachypodium distachyon*
*THRUMIN1* (AT1G64500)	Putative actin binding protein that connects phototropins with actin cytoskieleton at the plasma membrane (Whippo et al. 2011)	PREDICTED: XP_014751955.1 *E* value: 2e−36 Identity: 48%
*WEB1* (AT2G26570)	Proposed to be a cp-actin regulator, co-localizes with pmi2, no direct evidence that it binds actin (Kodama et al. 2010)	PREDICTED: XP_003562667.1 *E* value: 0.0 Identity: 51%
*JAC1* (AT1G75100)	Contains an auxilin-like J-domain. Probably cochaperones of the Hsp70 (Suetsugu et al. 2005, 2010a)	PREDICTED: XP_014753045.1 E value: 1e−42 Identity: 59%
*KAC1* (AT5G10470)	Kinesin-like proteins. Proposed to regulate cp-actin. They neither interact with microtubules nor have ATPase activity; no actin binding domain; one indication that KAC1 may interact with actin (Suetsugu et al. 2010b)	–
*KAC2* (AT5G65460)		PREDICTED XP_003564094.1 *E* value: 0.0 Identity: 64%
*PMI1* (AT1G42550)	Involved in chloroplasts and nucleus movements. Believed to regulate cp-actin (DeBlasio et al. 2005; Suetsugu et al. 2015)	PREDICTED: XP_003576813.1 E value: 0.0 Identity: 45%
*PMI2* (AT1G66840)	Possess long coiled-coil domain, co-localizes with WEB1. Proposed to be a cp-actin regulator (Kodama et al. 2010)	PREDICTED: XP_003569600.1 *E* value: 8e−41 Identity: 31%
*CHUP1* (AT3G25690)	Chloroplast outer membrane protein, binds actin and profilin, responsible for chloroplast positioning (Oikawa et al. 2008; von Braun and Schleiff 2008)	PREDICTED: XP_003567839.1 *E* value: 0.0 Identity: 63%
*RPT2* (AT2G30520)	RPT2 and NCH1 are localized at the plasma membrane and interact with phototropins; act redundantly to mediate chloroplast accumulation (Suetsugu et al. 2016)	PREDICTED: XP_003577852.1 *E* value: 1e−133 Identity: 41%
*NCH1* (AT5G67385)		PREDICTED: XP_003558544.1 *E* value: 0.0 Identity: 54%
*PP2A*-*2* (AT1G59830)	PP2A dephosphorylates phot2 by A1 subunit (Tseng and Briggs 2010; Sztatelman et al. 2016); A-2 subunit is involved in avoidance response (Wen et al. 2012; not confirmed by Sztatelman et al. 2016)	PREDICTED: XP_003563712.1 *E* value: 0.0 Identity: 92%

Light effects on phototropin expression were investigated in pea, rice, maize and Arabidopsis. Elliott et al. (2004) investigated phototropin expression in pea seedlings under the influence of red light using Northern blot. Only phototropin1, in two forms, occurs in pea. They are *PsPHOT1A* and *PsPHOT1B*, the latter one was assumed to be the main form. Under red light of 10 µmol m^−2^ s^−1^ the transcript level of *PsPHOT1A* did not change while *PsPHOT1B* was downregulated. The authors concluded that this downregulation was driven by phytochromes. No consistent blue-light effect was reported. In rice etiolated coleoptiles (Jain et al. 2007) the *OsPHOT1* gene was downregulated under blue (10 µmol m^−2^ s^−1^) and white light (75 µmol m^−2^ s^−1^), whereas the transcript level in leaves did not change. *OsPHOT2* gene was upregulated both in coleoptiles and leaves under the same light conditions. These results were obtained using real time PCR. Also in maize *ZmPHOT1* was observed to have a much higher expression in etiolated coleoptiles than *ZmPHOT2*. Exposure to blue light decreased *ZmPHOT1* gene expression while *ZmPHOT2* expression did not change much. Lower but more uniform expression of both *ZmPHOT1* and *ZmPHOT2* was demonstrated in young leaves using semi-quantitative real time PCR. No substantial changes were observed in leaves after blue-light irradiation (Suzuki et al. 2014). It is difficult to compare the results obtained by other groups with ours because of different culture conditions, leaf age and fluence rates used for sample irradiation.

A detailed study on phototropin expression during development, from seedlings to senescing leaves, was carried out on Arabidopsis using real time PCR (Łabuz et al. 2012). During the lifecycle of Arabidopsis *PHOT1* was downregulated and *PHOT2* upregulated when the plants were irradiated with white light of 100 or 120 µmol m^−2^ s^−1^. Analogous regulation was observed in mature leaves under blue and red light of 40 µmol m^−2^ s^−1^. Additionally, using null mutants *cry1*, *cry2*, *cry1cry2*, *phyA*, *phyB*, *phyAphyB*, photoreceptors involved in the regulation of phototropin mRNA level were postulated. The expression of *PHOT1* was shown to depend on cry1 and phyB, and the expression of *PHOT2*—on both cry1 and cry2 as well as phyA.

Our experiments on Brachypodium phototropin genes show regulation of the expression by light opposite to that in Arabidopsis. While in Arabidopsis, strong blue or red light downregulates *AtPHOT1* expression and upregulates *AtPHOT2* expression, in Brachypodium the same irradiation upregulates the expression of *BradiPHOT1* and downregulates the expression of *BradiPHOT2*. At present we cannot explain this discrepancy, but we believe two explanations should be considered. Firstly, it is possible that phototropin genes may have opposite functions in Arabidopsis and Brachypodium. This hypothesis may be easily tested by studying phototropin mutants in Brachypodium, what we plan to do in the future. An analysis of weak and strong blue-light controlled processes in single mutants of both phototropins in Brachypodium should clearly indicate any functional differences compared to Arabidopsis genes. The second explanation originates from the comparative analysis of the motif composition of phototropin promoter regions in Arabidopsis and Brachypodium. The results of this analysis are shown in Supplementary Table 2 and point to a high degree of similarity in the promoter regions since 53% of light-regulated motifs are shared across all promoters analyzed. As the light-dependent expression patterns seem to be inverted for *BradiPHOT1* and *BradiPHOT2* in comparison to the respective Arabidopsis genes, we analyzed cis-elements, looking for motifs that are shared between *AtPHOT1* and *BradiPHOT2* promoters, and between *AtPHOT2* and *BradiPHOT1*. We found one candidate motif, SORLIP2AT, to be present in *AtPHOT1* and *BradiPHOT2* but absent in *AtPHOT2* and *BradiPHOT1*. SORLIPs (Sequences Over-Represented in Light-Induced Promoters) are believed to be involved in the light activation of phyA-regulated genes in Arabidopsis (Hudson and Quail 2003). The family consists of five sequences (from SORLIP1 to SORLIP5) in Arabidopsis. It was shown that double SORLIP1 is required for high light induction of *ELIP* (Early Light-Induced Proteins) genes in Arabidopsis (Alvarez-Canterbury et al. 2014). Duplication and triplication of a SORLIP1 motif present in the promoter of the light-inducible protein gene (*LIP*) of *Dunaliella* dramatically increased its high light response (Baek et al. 2016). SORLIP motifs might be responsible for the observed light-expression pattern of phototropins in Brachypodium. On top of the SORLIP2AT motif presence being shared between *AtPHOT1* and *BradiPHOT2* promoters, we also found another SORLIP family motif, SORLIP1AT to be present in PHOT2 promoters for both Arabidopsis and Brachypodium and a SORLIP5AT motif only in *AtPHOT1* (see Table S2, positions 11-13). Further investigation is needed to check whether the observed differences in the number or ratio of these motifs, known to be over-represented in the phyA-induced promoters, are responsible for the opposite regulation of phototropin expression by light in the two species compared. It also remains to be investigated whether this regulation in Brachypodium is characteristic of other C3 grasses.

In summary, our work clearly demonstrates that chloroplast movements can be observed in a range of C3 grasses. We show that the process is likely to be controlled by phototropins in the analyzed species. To extend this analysis we investigated the influence of light on phototropin expression in a model grass species, *B. distachyon.* The similarity in chloroplast movements across grasses and the high homology of phototropin sequences of Brachypodium and those of other grass species analyzed suggests that Brachypodium may be a good model to study light driven chloroplast relocation in economically important Monocotyledones.

## Electronic supplementary material

Below is the link to the electronic supplementary material.

Supplementary material 1 (DOCX 169 kb)
