# Supplementary material for: Chloroplasts in C3 grasses move in response to blue-light
Source: Plant Cell Rep. 2020 Jul 13;39(10):1331–43. doi: 10.1007/s00299-020-02567-3 (PMC7497455; doi:10.1007/s00299-020-02567-3)
Supplement: Supplementary file 1 — Supplementary material 1 (DOCX 169 kb) [file 299_2020_2567_MOESM1_ESM.docx]

**Supplementary information**

Chloroplasts in C3 grasses move in response to blue-light

Weronika Krzeszowiec*, Maria Novokreshchenova, Halina Gabryś


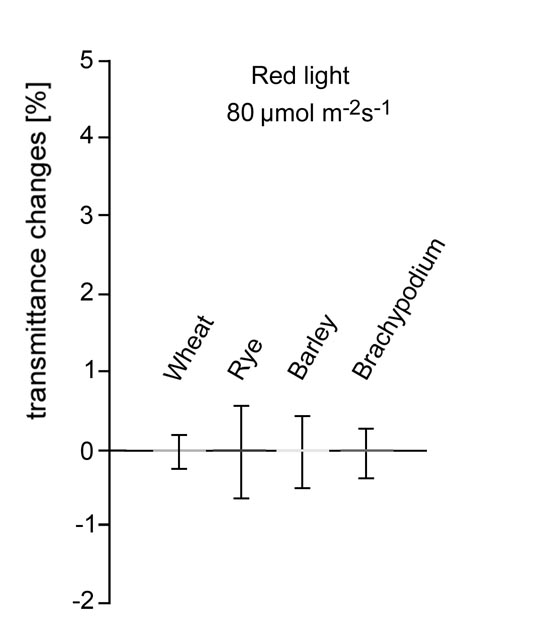


Fig. S1 Effects of red light on leaf transmittance in four species, wheat (*Triticum aestivum*), rye (*Secale cereal*), barley (*Hordeum vulgare*) and *Brachypodium distachyon*. Transmittance drifts following 90 min of continuous red light.


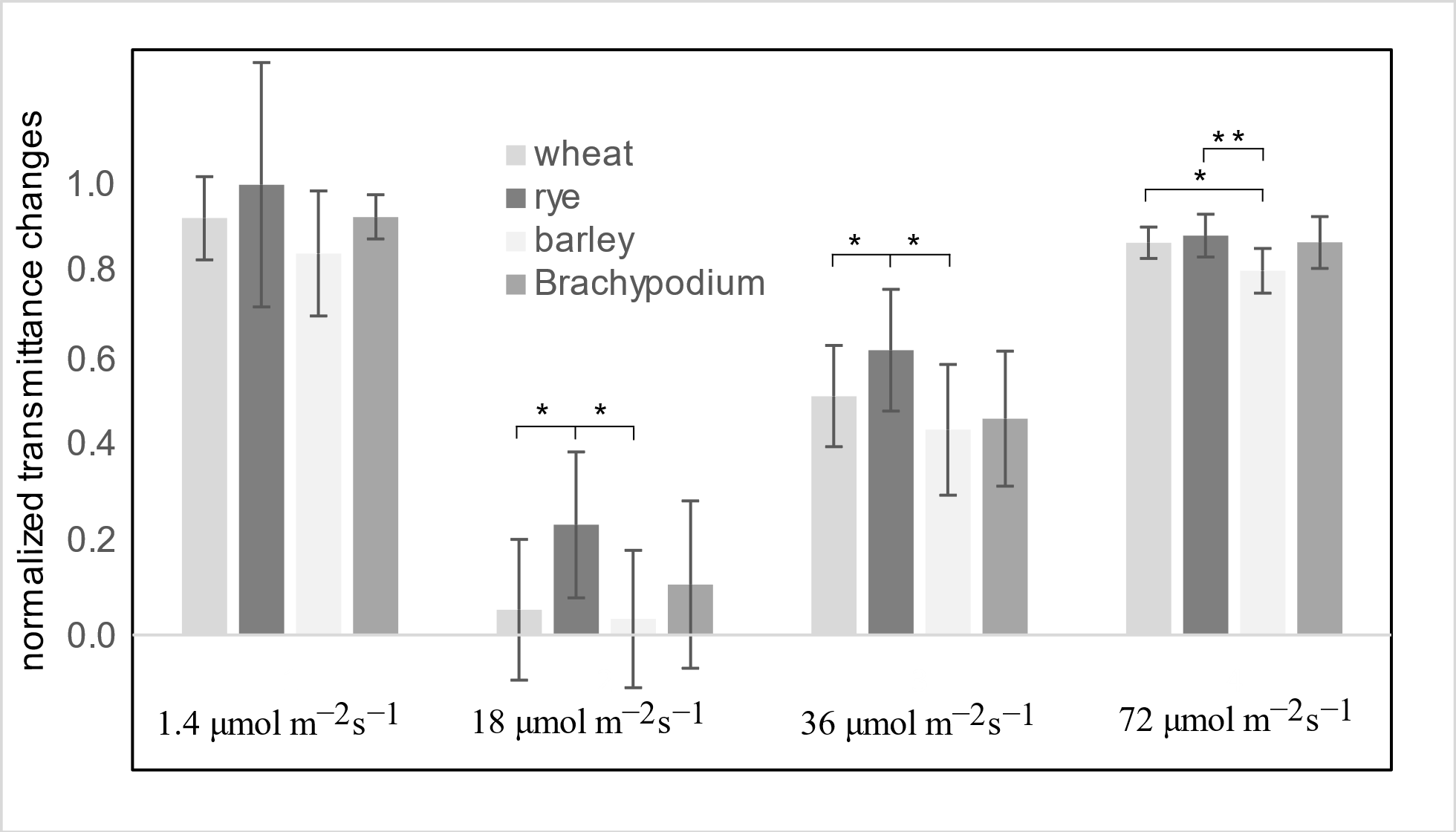


Fig.S2 Comparison of chloroplast response parameters for the investigated C3 grass species. Transmittance amplitudes were measured at consecutive steps of the recorded fluence-response curves. For each curve the amplitudes were normalized to the maximum transmittance change corresponding to full accumulation or avoidance chloroplast response. The statistical analysis was performed using one-way ANOVA and Tukey's test (GraphPad Instat3).

Table S1. Protein sequences used for phylogenetic analysis.

| ***PHOT1* gene** | | |
| --- | --- | --- |
| **Species** | **Annotated name** | **Source** |
| *Arabidopsis thaliana* | AT3G45780 | TAIR |
| *Brachypodium distachyon* | Bradi4g45310 | Phytozome |
| *Brachypodium stacei* | Brast05G300800 | Phytozome |
| *Brassica napus* | XP_013672833 | NCBI |
| *Brassica oleracea* | XP_013628772 | NCBI |
| *Camelina sativa* | XP_010426007 | NCBI |
| *Capsella grandiflora* | Cagra.1884s0002 | Phytozome |
| *Eucalyptus grandis* | Eucgr.J02109 | Phytozome |
| *Gossypium hirsutum* | XP_016665229 | NCBI |
| *Hordeum vulgare* | KAE8791225 | GenBank |
| *Musa acuminata* | GSMUA_Achr4G30620_001 | Phytozome |
| *Nicotiana tabacum* | XP_016436377 | NCBI |
| *Oryza sativa* | Os11g01140 | Phytozome |
| *Panicum hallii* | Pahal.B02829 | Phytozome |
| *Panicum virgatum* | Pavir.J02766 | Phytozome |
| *Selaginella moellendorffii* | 230655 | Phytozome |
| *Setaria italica* | Seita.8G001400 | Phytozome |
| *Setaria viridis* | Sevir.8G020400 | Phytozome |
| *Spirodela polyrhiza* | Spipo23G0029400 | Phytozome |
| *Tarenaya hassleriana* | XP_010535562 | NCBI |
| *Triticum aestivum* | SPT18078 | GenBank |
| ***PHOT2* gene** | | |
| *Arabidopsis thaliana* | NP_001318824 | NCBI |
| *Brachypodium distachyon* | Bradi5g07360 | Phytozome |
| *Brachypodium stacei* | Brast09G048300 | Phytozome |
| *Brassica napus* | XP_013716737 | NCBI |
| *Brassica oleracea* | XP_013608703 | NCBI |
| *Camelina sativa* | XP_010483373 | NCBI |
| *Capsella grandiflora* | Cagra.0891s0009 | Phytozome |
| *Eucalyptus grandis* | Eucgr.I01551 | Phytozome |
| *Gossypium hirsutum* | XP_016716710 | NCBI |
| *Hordeum vulgare* | KAE8791225 | GenBank |
| *Musa acuminata* | GSMUA_Achr4T30620_001 | Phytozome |
| *Nicotiana tabacum* | XP_016471106 | NCBI |
| *Oryza sativa* | XP_015620213 | NCBI |
| *Panicum hallii* | Pahal.G01546 | Phytozome |
| *Panicum virgatum* | Pavir.J34029 | Phytozome |
| *Selaginella moellendorffii* | 230655 | Phytozome |
| *Setaria italica* | Seita.7G046200 | Phytozome |
| *Setaria viridis* | Sevir.7G051600 | Phytozome |
| *Spirodela polyrhiza* | Spipo23G0029400 | Phytozome |
| *Tarenaya hassleriana* | XP_010518756 | NCBI |
| *Triticum aestivum* | SPT18078 | GenBank |

Table S2. Analysis of promoter regions of *PHOT1* and *PHOT2* in *Arabidopsis thaliana* and *Brachypodium distachyon* using Database of Plant Cis-acting Regulatory DNA Elements (PLACE). All descriptions taken from the database.

| ­­ | Accession number | Genes | Site name | Consensus sequence | Binding factor | Family | Function |
| --- | --- | --- | --- | --- | --- | --- | --- |
| 1 | S000024 | BradiPhot1, ATPhot2, BradiPhot2 | ASF1MOTIFCAMV | TGACG |  |  | "ASF-1 binding site" in CaMV 35S promoter; ASF-1 binds to two TGACG motifs; found in HBP-1 binding site of wheat histone H3 gene; TGACG motifs are found in many promoters and are involved in transcriptional activation of several genes by auxin and/or salicylic acid; may be relevant to light regulation; binding site of tobacco TGA1a; TGA1a and b show homology to CREB; TGA6 is a new member of the TGA family; abiotic and biotic stresses differentially stimulate "as-1 element" activity. |
| 2 | S000144 | ATPhot1, BradiPhot1, ATPhot2, BradiPhot2 | EBOXBNNAPA | CANNTG | KW napA; storage protein; ABRE; E-box; seed; |  | Cis-acting elements RT recognized by R2R3-MYB, BZIP, and BHLH factors control RT light-responsive and tissue-specific activation of RT phenylpropanoid biosynthesis genes; E-box of napA storage-protein gene of *Brassica napus*; This sequence is also known as RRE (R response element) (Hartmann et al., 2005). |
| 3 | S000198 | ATPhot1, BradiPhot1, ATPhot2, BradiPhot2 | GT1CONSENSUS | GRWAAW | GT-1; light | TATA | Consensus GT-1 binding site in many light-regulated genes, e.g., RBCS from many species, PHYA from oat and rice, spinach RCA and PETA, and bean CHS15; R=A/G; W=A/T; for a compilation of related GT elements and factors see Villain et al. (1996); GT-1 can stabilize the TFIIA-TBP-DNA (TATA box) complex; the activation mechanism of GT-1 may be achieved through direct interaction between TFIIA and GT-1; binding of GT-1-like factors to the PR-1a promoter influences the level of SA-inducible gene expression. |
| 4 | S000199 | ATPhot1, BradiPhot1, ATPhot2, BradiPhot2 | IBOXCORE | GATAA | I box; I-box; rbcS; light regulation; light; leaf; shoot |  | "I box"; conserved sequence upstream of light-regulated genes of both monocots and dicots. |
| 5 | S000203 | ATPhot1, BradiPhot1, ATPhot2, BradiPhot2 | TATABOX5 | TTATTT |  | TATA | Cis elements and trans-acting factors affecting regulation of a RT nonphotosynthetic light-regulated gene for chloroplast glutamine RT synthetase;  TATA box found in the 5'upstream region of pea (*Pisum* *sativum*) glutamine synthetase gene; a functional TATA element by in vivo analysis. |
| 6 | S000362 | ATPhot1, BradiPhot1, BradiPhot2 | REALPHALGLHCB21 | AACCAA |  |  | “REalpha” found in *Lemna gibba* Lhcb21 gene promoter; Located at -134 to -129; binding site of proteins of whole-cell extracts; the DNA binding activity is high in etiolated plants but much lower in green plants; required for phytochrome regulation. |
| 7 | S000383 | BradiPhot2 | TBOXATGAPB | ACTTTG | GAPB; glyceraldehyde-3-phosphate dehydrogenase; light-activated KW transcription |  | "Tbox" found in the *Arabidopsis thaliana* GAPB gene promoter; located between -94 and -89 (T1) and also between -84 and -79 (T2); mutations in the "Tbox" resulted in reductions of light-activated gene transcription; GAPB encodes the B subunit of chloroplast glyceraldehyde-3-phosphate dehydrogenase(GADPH) of A.t. |
| 8 | S00039 | ATPhot1, BradiPhot1, ATPhot2, BradiPhot2 | GATABOX | GATA | ASF-2; GATA box; Cab; chlorophyll a/b binding protein; leaf; KW shoot; |  | "GATA box"; GATA motif in CaMV 35S promoter; binding with ASF-2; three GATA box repeats were found in the promoter of Petunia (P.h.) chlorophyll a/b binding protein Cab22 gene; required for high level, light regulated, and tissue specific expression; conserved in the promoter of all LHCII type I Cab genes. |
| 9 | S000392 | ATPhot1, BradiPhot1, ATPhot2, BradiPhot2 | -10PEHVPSBD | TATTCT | psbD; chloroplast gene expression; circadian rhythms; light KW regulation; |  | "-10 promoter element" found in the barley (H.v.) chloroplast psbD gene promoter; involved in the expression of the plastid gene psbD which encodes a photosystem II reaction center chlorophyll-binding protein that is activated by blue, white or UV-A light. |
| 10 | S000395 | ATPhot1, BradiPhot1, ATPhot2, BradiPhot2 | INRNTPSADB | YTCANTYY | initiater; light-responsive transcription; TATA-less promoter; KW psaDb; Inr element; |  | "Inr (initiator)" elements found in the tobacco psaDb gene promoter without TATA boxes; light-responsive transcription of psaDb depends on Inr, but not TATA box. |
| 11 | S000482 | ATPhot2, BradiPhot2 | SORLIP1AT | GCCAC | phyA; phytochrome; light; |  | One of the Sequences Over-Represented in Light-Induced Promoters (SORLIPs) in Arabidopsis; computationally identified phyA-induced motifs; SORLIP 1 is most over-represented and most statistically significant. |
| 12 | S000483 | ATPhot1, BradiPhot2 | SORLIP2AT | GCCAC | phyA; phytochrome; light; |  | One of the Sequences Over-Represented in Light-Induced Promoters (SORLIPs) in Arabidopsis; computationally identified phyA-induced motifs. |
| 13 | S000486 | ATPhot1 | SORLIP5AT | GAGTGAG |  |  | One of the Sequences Over-Represented in Light-Induced Promoters (SORLIPs) in Arabidopsis; computationally identified phyA-induced motifs; over-represented in both light-induced cotyledon-specific and root-specific genes (Jiao et al. 2005). |
| 14 | S000487 | BradiPhot2 | SORLREP2AT | ATAAAACGT | phyA; phytochrome; light; |  | one of the Sequences Over-Represented in Light-Repressed Promoters (SORLREPs) in Arabidopsis; Computationally identified phyA-induced motifs;. |
| 15 | S000506 | BradiPhot1, BradiPhot2 | PRECONSCRHSP70A | SCGAYNRNNNNNNNNNNNNNNNHD |  |  | Consensus sequence of PRE (plastid response element) in the promoters of HSP70A in Chlamydomonas; Involved in induction of HSP70A gene by both MgProto and light; S=G/C; Y=C/T; R=A/G; H=T/C/A; D=A/T/G. |
